# Abstracts and Gleanings

**Published:** 1878-10-20

**Authors:** 


### *ABSTRACTS AND GLEANINGS.*

### PROCEEDINGS IN FRENCH SOCIETIES.

Academy of Medicine, Paris.—*Session July* 25, 1878.—M. E. Lancereaux presented a paper which had been read before the International Geographical Congress, 1875, entitled:

*Geographical Distribution of Pulmonary Phthisis.*—The disease is found in all climates and among all people, but it is not equally destructive everywhere. Relatively rare in polar regions, the disease prevails more especially in temperate climates, and remarkably so in dense populations, and particularly in great industrial centers. It is of common occurrence in the tropics too where its course is very rapid. But these general notices only give a vague idea of cosmical influences in the production of phthisis. In order to arrive at an exact conclusion on the ethiological conditions of the disease, he had been obliged to analyse, in a wide sense, climate in its chief elements, temperature, moisture, dryness, altitude, etc., and to take into account the habits, way of living, muscular activity of different communities and tribes, and he had arrived at the following conclusions: cold has no influence in the genesis of tuberculosis; the inhabitants of elevated regions, 800 to 1,000 yards above the level of the sea, are as exempt as those living in polar latitudes. On the contrary those inhabiting low, damp and warm districts are very subject to phthisis. Crowded quarters, poor ventilation, with food unsuited to the climate, excess in alcoholic beverages, and lack of muscular exercise are circumstances which most favor the development of tubercle. Savags tribes do not suffer from this disease which decimates the civilized races; hence this conclusion, *that tubercular phthisis is a disease of civilization*, and it is the duty of the State to procure its destruction. To attain this, Doctor Lancereaux demands laws regulating the construction of houses, increased breadth of streets and abundant air space, not only for soldiers in barracks, but for workmen in factories, families in tenement houses, children in schools, students in colleges and all those in the humblest positions.—*Lancet and Clinic.*

### PORRO'S OPERATION; ANOTHER SUCCESSFUL CASE.

M. Wasseige reported to the *Academie royale de Belgique* a completely successful operation of Porro, prefacing the report with a history of the operation and a comment of comparison between it and the Cæsarean section. The anthor claimed that only two cases had resulted fatally after this operation, while it is probable that all the patients would have died after Cæsarean section. We give below a very brief account of the case and the operation :

Josephine Moyse, rachitic, hight 1. 2. m., sacro-pubic diameter 0.04 m., marked diminution of all the diameters of the pelvis ; was examined during the seventh month of pregnancy and directed to present herself at the hospital at the intimations of labor. She entered during the service of M. Wasseigo, April 14th, 1878, at 6½ o'clock P. M., having been in labor already three hours.

The operation was performed at nine o'clock p. m., in the presence M. Vaust, director of the Maternite, M M. Plucker, Closon and Dexhamps, assistants, and Drs. Lebeau and Bidlot with the entire *cortege* of the antiseptic method of Lister. The rectum and bladder were emptied by injection and catheterization, and the patient was put thoroughly under chloroform before the operation was commenced.

M. Wasseigo now made an incision descending from the umbilicus to 0.04 m. above the symphisie pubis. The uterus having been uncovered, the incision was completed over the index finger with a blunt pointed bistoury. The uterus was then opened* and the fœtus rapidly extracted. The child, a female, was full of life. The operator now raised the uterus with its adnexa out of the pelvic cavity by traction upon the incised edges. The placenta fell forwards and was removed. The uterine incision was now seen to run along the lower half of the anterior face of the organ, a situation accounted for by the position of the uterus elevated in the abdominal cavity on account of the contraction of the pelvis. The ecraseur was now adjusted about opposite to the internal os in order to have the whole uterine wound above the chain when the entire organ was cut off with a long bistoury, without the escape of even a drop of blood. The ecraseur was then used as a clamp and the bistoury was passed across, dividing everything 0.02 m. above the chain.

*Porro's operation, as since improved, opens the uterus only after its ablation from the pelvic cavity, an improvement whose advantage is obvious enough.—Trans.

The uterine stump formed a veritable rose at the lower angle of the uterine section. A long, strong needle was passed through it to prevent its retraction within the abdominal cavity. The chain of the ecraseur being now removed, a drainage tube was inserted into the cul-de-sac of Douglas ; the toilet of the peritonium was completed and the abdominal wound was closed at four points with metallic suture *enchevillee* and a whole series of superficial sutures of silver wire.

The patient was then bathed with a carbolized solution and the uterine stump was cauterized with a solution of chloride of zinc eight times. The ligature chain about the stump, the needle and the stump itself were enveloped in the Lister dressing and two caoutchouc sacs filled with ice were placed upon the abdomen. The time occupied during the operation and the dressing was one hour.

The child weighed 2,580 grammes, and was 48½ centimeters in length. The uterus and its adnexa weighed 765 grammes.

The mother recovered perfectly and left the hospital with a healthy child, May 24, forty days after the operation.

Details of the case and operation will be published in the next number of the Bulletin of the Academy—*Gazette Hebdomadaire*, June 7th, 1878.—*Clinic.*

---

### TINC. IODINE IN FEVERS.

---

Dr. Laughlin (in *Lancet and Clinic*) says, "I have extended its use to the treatment of remittent and the so-called typho-malarial fevers in connection with other appropriate remedies, with good effect. Its property of relieving nausea and vomiting makes it very valuable in cases of remittent attended with these conditions. I feel confident that it aids materially in cutting short remittent fever.

In chronic ague it would seem to fulfill a double indication by reducing the enlarged spleen, and at the same time arresting the pyrexia. Latterly I have used and prefer Lugol's solution to the tincture, as it is in a condition to mix readily with water—with which it should always be largely diluted—and is somewhat less liable to decomposition by long standing; giving it in doses of ten to twelve drops three times a day in ague, and five to eight drops every six hours in fever without regard to pyrexia; while watching its effects upon the schneiderian and pharyngeal mucous membrane. Dr. Munro gave the tincture in from fifteen to twenty-five drop doses three times a day. In cases of chronic malarial cachexia where a combination of iron, quinine, arsenic and strychnine are indicated, I have found the combination with iodine or its simultaneous administration of great importance in the reduction of the enlarged spleen.

### THE AMBULATORY TREATMENT OF PSORIASIS VULGARIS.

The author says that, as is known, the treatment of psoriasis is a difficult and thankless task. By the greatest patience and perseverance only temporary abatement is attained, and not rarely a new and more intractable eruption follows; of the agents hitherto employed, some are not without danger from long use, as arsenic; some are too troublesome, as baths, inunctions, etc.; some too painful, as friction with brushes, Vleminck's solution, etc.; and finally, the greater part are without effect. It is therefore no wonder that even the most eminent authorities include this among the incurable skin diseases.

The above facts, the author thinks, justify him in publishing a method of treatment without danger, cleanly and cheap. With the prospect of a cure, intelligent patients can not only be treated without confinement, but pursue the treatment without the oversight of the physician.

Crystalized carbolic acid dr ij is dissolved in ozj of collodion, and brushed over the affected portions of skin. When the eruption is limited it may all be brushed over at once, but where it is extensively diffused, a gradual application is preferable, say one day an arm, and after two or three days the other arm, or foot, etc. This precaution should be observed to prevent carbolic acid poisoning since it readily passes from the skin into the blood. The application is not repeated as long as the collodion remains fixed to the skin. It is superfluous to put on fats. The physician should apply the solution, for often a patient makes a misuse of medicaments.

In a few minutes after the painting pain arises, but it is endurable, and does not continue more than ten or fifteen minutes. In a few days the patient and physician will be surprised to find that the eruption has lost its original character. Instead of the nasty, red, fissured, bleeding appearance there are seen smooth shining flat collodion flecks which are not disturbed by washing, baths, or friction of the clothes.

G. has also found that psoriasis spots not brushed, sometimes disappear, as it seems, from absorption of the carbolic acid. His observation has noted no outbreak of the eruption in new places as occurs with other methods of cure.

The application must continue as long as the seat of the eruption is red and infiltrated. The treatment on the whole may require 2, 3 or more months, but when the convenience and ease are considered this will not appear long.

Concerning relapses they need excite no apprehension, since they again give way under the same treatment.—*Lancet & Clinic.*

### RECENT PROGRESS IN PATHOLGICAL ANATOMY.

BY R. H. FITZ, M. D.

[From the Boston Medical and Surgical Journal.]

### PATHOLOGICAL ANATOMY.

Regeneration of Nerves.—Gluck has recently conducted a series of experiments with reference to the healing of nerves after they have been cut. The sciatic nerve of fowls and the pneumogastric of rabbits were exposed and cut through, the results of the operation depending upon the subsequent relation of the cut ends to each other.

Immediately after the section was made, the nerve fibres projected beyond the retracted sheath, and the myeline escaped. The cut ends were united during the next few days by a grayish white translucent tissue. If a considerable portion (one centimetre or more) of the nerve was removed, the intervening gray tissue became converted into a dense fibrous callous, no regeneration of the nerves occurred, permanent paralysis resulted, and the animals died during the subsequent five months. When, however, the cut ends were closely united, without the removal of a portion of the nerve, the results were quite different, being the more favorable the less the displacement. In certain cases, where the nerve was simply perforated, longitudinal rows of fusiform cells, surrounded by abundant homogeneous intercellular substance, were found within seventy-two hours after the operation. These bridged over the interval between the cut ends, sometimes extending from a central to a peripheral fibre. After eight days the ends were united by non-medullated nerve fibres, which slowly and gradually became thicker.

When the nerve was wholly cut across, and the ends united by sutures, the healing process took place in a similar manner, more time being required. Within eighty hours after the operation the wound was closed by a gray granulation tissue, in which, within a fortnight, spindle cells arose, apparently from the nuclei of the neurilemma, and served to unite the cut axial fibres, a differentiaton into axis-cylinder and myeline apparently took place later within these cells.

The author considers that the newly-formed fibres arise from these large granular spindle cells, which are to be regarded as of new formation rather than as outgrowths from pre-existing fibres. They resemble ganglion cells rather than those of connective tissue.

The results of the histological examination were confirmed by physiological experiment, the time of the return of the function to the nerve trunks corresponding with the appearances observed under the microscope.—*Bost Med. and Sur. Jour.*

### AIDS TO LABOR.

Dr. Spalding in *Detroit Academy of Medicine* in opposing abuse of the forceps remarks:

What other means can be employed to diminish the time of labor, and thereby its suffering? As prominent in efficacy among such means, I would enumerate: 1st. Firm and persistent pressure, made upon the uterine neck *with every pain.* 2d. The application of belladonna to the uterine neck. 3d. The early rupture of the membranes. 4th. The proper use of uterine motor stimulants. 5th. A proper regard for the position of the patient

I am in the habit of regarding a labor as fairly commenced when with uterine pains the uterine neck is dilated to the size of a quarter dollar.

My experience has made me positive, that from this time forward, by the employment of the measures above indicated, the duration of the first stage of labor can be diminished *at least one half.* I would therefore stimulate uterine action by making decided pressure with the finger upon the uterine neck upward under the public arcade, with every pain, which has the effect not only to increase most decidedly the force of the pains, but not unfrequently in cases where there is marked inactivity of the uterus, pains can be excited at will by this procedure. Belladonna also is of undoubted utility in assisting relaxation, and thereby facilitating dilatation of the uterine neck. If belladonna ointment be used to lubricate the finger we secure thereby its continuous effect. If now as soon as the os has become dilated to the size of a half dollar, the amniotic liquid be evacuated by rupture of the membranes, the uterus will after a few moments of rest, contract with increased force, its previous distention preventing its complete action, just as a bladder is semi-paralyzed by over distention.

Should there exist a degree of uterine inertia calling for farther stimulation, we may now administer a uterine motor stimulant, perhaps a cup of very strong coffee, which has this effect; even a drink of ice-water will have the effect. Alcoholic stimulation will almost always do it; but undoubtedly the most reliable agents, yet ascertained, are quinine and the old stand-by, secale cornutum or ergot of rye, which I am free to admit, I am in the habit of using. I am aware that there is a strong antipathy in the minds of many against the use of ergot; they claiming that there is danger of lacerating the perineum, and causing still-births as the result of too violent pains produced.

I simply reply that it is no argument against the proper use of morphine, for instance, that while you were endeavoring to overcome an attack of colic it would be possible to give enough to kill your patient. You are supposed to give it in *proper* and *safe* doses, and so with ergot. Squibb's fluid extract of ergot I have found generally reliable, if fresh. If two thirds of a teaspoonful is given it will act in from one-half to three-fourths of an hour; if not it may be safely repeated. My rule is then, give enough to produce the desired effect and then stop.

I mentioned also position of the patient as a point to be looked after, for it will sometimes happen with a patient lying for instance in a twisted position, half upon the back and half upon the side, that rotation of the head in the pelvic concavity will be prevented and the action of the uterus for a long time rendered unavailing, a condition of things which a change of position will rectify at once.

It will be seen that by the employment of the above means we have rendered valuable assistance to our patient *from the very commencement of her labar*, and it is not until now, when the os uteri is fully dilated, that it is possible to render any assistance whatever with the forceps. But the previous and continuous stimulation of the uterus which we have excited will be continued, and unless there be a serious disproportion between the head of the child and the pelvis of the mother the remainder of her labor will be very brief. So that unless adjustment of a malposition is required, or a disproportion exists, or the occurrence of some complication, as convulsions, exhaustion, or hemorrhage, rendering a few minutes valuable, the use of the forceps is unnecessary.

In verification of what I have claimed I may be permitted to state that in the last 100 consecutive cases of confinement, which I have conducted, I have not used the forceps once, although encountering the same variety of cases as are ordinarily considered as justifying the use of the forceps.

Furthermore, the *average time* in which I have been engaged is less than *two* hours, the longest being four hours.

Mr. President—While my own experience has proven to my satisfaction the truth of the principles herein stated, I have laid no particular claim to originality. I have simply attempted to drag up from their dormant slumber a few practical truths, already well known but unused to the extent which they deserve.

Let us not, when dealing with life, death and suffering, allow our fondness for operating to prevent us from giving our patients the benefit of *all* that is known to be practical for the relief of their suffering.

Conservative surgery is the order of the day; we no longer estimate a surgeon's skill by the number of limbs which he has sacrificed, but rather by the number saved. If this is an indication of progress in surgery, how long must we wait for obstetricians to fall into line and cease to pride themselves upon their increasing ratio of forceps deliveries?—*Detroit Lancet.*

### ETHER AND CHOLOFORM IN PUERPERAL CONVULSIONS.

*To the Editor of The Medical Record.*

SIR:—Supplementary to what I have said in your issue of Sept. 14th on the anæsthetic treatment of puerperal convulsions, I wish to put on record the following testimonials:

Dr. John Crowell, Haverhill Mass., in a letter to me, dated September 13, 1878, says:

"Judging from my own experience, many more patients are saved by anæthesia than by the old methods adopted in my early practice."

Dr. G. W. Garland, Lawrence, Mass., writes that he considers "the anæsthetic treatment as invaluable, and cannot be dispensed with." He has, however, never treated a case without other remedies.

From Dr. Enoch Cross, the oldest physician in Newburyport, now nearing his eighth decade:

"I have had experience in all kinds of treatment of that dire disease. In my earlier years I used to witness disastrous results, but since I have trusted to ether, have had uniform success."

Dr. F. A. Howe, Newberyport: "Full and continious anæsthesia alone can be relied on in urgent cases." Dr. Howe, who has had many cases, trusts mainly to ether, or if he could not obtain ether, would use chloroform. He also uses, as adjuncts, chloral hydrate and the bromides.

Dr. J. A. Douglass, of Amesbury, claims good results from veratum in large doses.

From Dr. Gilman Kimball, of Lowell, letter bearing date Sept. 13th:

"I have seen but one case treated with ether, and that seemed to show its good effect."

Dr. H. J. Cushing, of Merrimac, reports five cases. Two of them were fatal. In one no ether was used, and in the other it does not seem to have been administered preservingly. In the three cases that recovered ether was freely used.

Dr. S. J. Potter, Hamilton, Ohio. Dr. P. is an eclectic of repute in the West. (From a letter dated 14th inst.):

I employ chloroform and ether, of each equal parts, by inhilation, in the treatment of puerperal convulsions, and venesection, too, if the patient be plethoric. Chloroform is as safe as ether, and more prompt. I use these remedies in all cases in the above-mentioned proportions."

Dr. W. H. Kimball, of Andover, Mass. Dr. K. is President of our Essex North Medical Society. (Letter dated the 17th inst.):

"Have chiefly relied on inhilation of ether in treatment of puerperal convulsions for many years. I have more faith in this course than in any other."—*Medical Record.*

### BRYONIA AND DRASERA IN THE TREATMENT OF WHOOPING-COUGH.

Dr. Louvet-Lamare. (*L' Annee Medicale*, June, 1878, p. 105.)

The author gives in the first or catarrhal stage of the disease a daily dose of 1 gram of the tincture of bryonia to a child of seven years. The medicine does not abridge this stage, but it very sensibly diminishes the tracheo-bronchitis, which is then the only morbid manifestation; moreover, being bitter, it stimulates the appetite, and does not occasion nausea.

When the characteristic cough occurs, he gives the tincture of drasera in daily doses of 1 gram for a child of seven years, and the frequence and violence of the coughing fits at once diminish. The value of this treatment has been proved in a large number of cases, and also in the fits of coughing, in certain cases of bronchitis, and even in phthisis, though not so marked as in pertussis. But little success has been obtained from it in coughs caused by laryngitis and laryngo-tracheitis.

The dose may be increased by 5 drops or more every second day without danger, as it is not poisonous. The cough diminishes still more, but usually lasts for two or three weeks before convalescence. If, on the contrary it be given to a child who has had the cough for three weeks or so, one may expect to *cure the worst cases in a few days* From a large number of cases thus treated, the author concludes that the disease may be shortened to at least one half the time required by change of air, which is often impossible.—*Chicago Med. Journal.*

### TAPPING A VOMICA IN PHTHISIS.

Williams.—(*Br. Med. Jour.*, July 20, 1878.)

A patient aged twenty-eight, had had consumptive disease for six years. A large cavity with pleuritic adhesions had formed at the base of the left lung, besides others in other parts. The cough was harrassing, the expectoration very profuse (nearly a litre every 24 hours,) and very offensive. Microscopical examination showed that lung degeneration was rapidly advancing. At the writer's request, Mr. Erichsen introduced a large-sized trocar and cannula between the seventh and eighth ribs, and withdrew a litre of fœtid pus; the operation being somewhat difficult on account of the toughness of the fibrotic lung. Relief was immediate, all fœtor ceased, both pulse and temperature fell, and the expectoration was reduced to 60-100 C. C. Limited pneumothorax and cutaneous emphysema followed, the latter soon subsiding. Even at the expiration of a month, the discharge was slight. The cavity had several times been washed out with antiseptic fluids.

The operation had previously been done in this country by Dr. Pepper, in England by Dr. Ramadge, in Germany by Dr. Mosler, and by others. The practice is only suited to exceptional cases, such as those of fœtid expectoration. It has been recommended to accomplish the same purpose by catheterisation of the air-passages, by large thoracentesis, by aspiration, by caustic and by galvano-caustic, the latter preferred by Koch of Berlin

### A VEHICLE FOR QUININE.

Milk is recommended as a good solvent of quinine and is said to disguise its bitterness. One grain will dissolve in an ounce of milk, and render it scarcely bitter, while two grains do not make it markedly bitter. Further, five grains dissolved in two ounces of milk do not render it very unpleasant, while, put in a tumbler of milk, the bitterness all but disappears. Mr. Palmer, of the Birmingham General Dispensary, uses a solution of quinine in glycerine—one grain to one drachm. A dose of this can be given in a wineglassful of milk. Milk would seem to be a good vehicle in which to give quinine to children, but with regard to solubility it appears strange that so few doctors make use of the neutral sulphate of quinine, which is soluble in water without the addition of acid, and which, therefore offers every facility for administering quinine in a liquid form.—*Lancet & Clinic.*

### TREATMENT OF MAMMARY ABSCESS.

Dr. S. W. Gould, of Argosy, Ind., gives, in the *Med. and Sur. Reporter*, his treatment for threatened mammary abscess, under which, for a decade, he has not been obliged to lance a single breast. It consists in the application of chloroform and glycerine, equal parts. As the substances are of unequal weight, the phial containing them should be thoroughly shaken, the mixture quickly applied, and the part covered with oiled silk, or something equally impermeable, to prevent too rapid evaporation. So confident is he of the efficacy of this remedy that, after a ten years' trial of it, he is led to consider a gathered breast an evidence of professional neglect.—*Detroit Lancet.*

### PROCEEDINGS IN FRENCH SOCIETIES.

ACADEMY OF MEDICINE, PARIS.—*Session July* 25, 1888.—M. E. Lancereaux presented a paper which had been read before the International Geographical Congress, 1875, entitled:

*Geographical Distribution of Pulmonary Phthisis.*—The disease is found in all climates and among all people, but it is not equally destructive everywhere. Relatively rare in polar regions, the disease prevails more especially in temperate climates, and remarkably so in dense populations, and particularly in great industrial centers. It is of common occurrence in the tropics too where its course is very rapid. But these general notices only give a vague idea of cosmical influences in the production of phthisis. In order to arrive at an exact conclusion on the ethnologic l conditions of the di-ease, he had been obliged to analyse, in a wide sense, climate in its chief elements, temperature, moisture, dryness, altitude, etc., and to take into account the habits, way of living, muscular activity of different communities and tribes, and he had arrived at the following conclusions: cold has no influence in the genesis of tuberculosis; the inhabitants of elevated regions, 800 to 1,000 yards above the level of the sea, are as exempt as those living in polar latitudes. On the contrary those inhabiting low, damp and warm districts are very subject to phthisis. Crowded quarters, poor ventilation, with food unsuited to the climate, excess in alcoholic beverages, and lack of muscular exercise are circumstances which most favor the developement of tnbercle. Savage tribes do not suffer from this disease which decimates the civilized races, hence this conclusion, *that tubercular phthisis is a disease of civilization,* and it is the duty of the State to procure its destruction. To attain this, Doctor Lancereaux demands laws regulating the construction of houses, increased breadth of streets and abundant air space, not only for soldiers in barracks, but for workmen in factories, families in tenement houses, children in schools, students in colleges and all those in the humblest positions.—*Lancet and Clinic.*

### CÆSARAEN SECTION AFTER DEATH.

In the Obstetrical Society of Cincinnati, Dr. Cleveland reported a case of Cæsarean section performed, at least, one hour after death of the mother from convulsions, in which the child was saved.

He remarks that "the hypothesis that the woman was in a swoon, thus prolonging the life of the child, will not hold good in this case, for I know the mother was dead. It appears to me, when viability is limited to fifteen minutes or half an hour after maternal death, the well-known capacity of the fetus in resisting asphyxia is not taken fully into account, which, of course, will be increased by the residual oxygen that is in the placenta at the time of death."

### OXALATE OF CERIUM AND CAFFEIN AS PR[illegible]VENTATIVES OF THE NAUSEATING EFFECTS OF OPIUM.

BY SAMUEL C. BUSEY, M. D.

To Professor Da Costa is due the credit of having first suggested the combination of bromide of potassium with morphia to obviate the disagreeable after-effects which so frequently distress patients to whom morphia has been administered. It frequently happens that the potassium salt can not, in consequence of its bulk and unpleasant taste, be employed. Especially is it objectionable when the practitioner prefers to use opium either in the form of pill or powder; nor is its therapeutic effect always desirable.

To meet these objections I have, for some time past, combined the oxalate of cerium with opium, and have in very many instances found it equally efficatitious in obviating the nausea and mitigating the distressing after-effects. Sir James Simpson introduced the oxiate into use as a very valuable agent in relieving the nausea and v omiting of pregnancy. Since then, its employment has been very widely extended, and it has become quite common to combine it with drugs to secure thəir tolerance to the stomach.

For along time I have been accustomed to prescribe a strong docoction of coffee, without milk or sugar, in drachm doses administered every fifteen minuees, to relieve the nausea and headache following the employment of opium. Since the introduction of the effervescing citrate of caffein, I have found it a very agreeable and efficient substitute for the less palatable beverage. These agents are, however, only appplicable after the occurrence of the stomachie disturbances. The citrate of caffein, though less efficient than the oxalate of cerium, may be combined with opium. It is inadmissible in those cases where opium produces wakefulness instead of drowsiness. I have sometimes fancied that it lessened the soporific, without affecting the analgesic, properties of opium.

It may be that these applications of the drugs are quite common; but if they have been previously published, it has escaped my observation.—*American Practitiononer.*

---

### VINEGAR IN POST PARTUM HEMORRHAGE.

---

At the American Gynelogical Society Dr. Penrose read a paper on the above subject in which he states:

The remedy for the arrest of post-partum hemorraghe was *common vinegar.* Sincc 1854 he had used it with invarable success. The advantages claimed for it were:

1. That it could be readily obtained;
2. That it could be easily applied, and without special apparatus;
3. That it always cured the hemorrahge, or rather, it had not failed in his practice;
4. That it was sufficiently irritating to excite the most sluggish uterus to contraction, and yet not so irritating as to be subsequently injurious;
5. That it was an admirable antiseptic;
6. That it acted on the lining membrane of the uterus as an astringent.

Dr. Penrose used this remedy in the following manner: saturate a rag with vinegar, carry it into the cavity of the uterus, and squeeze it. In the vast majority of cases the hemorraghe ceased as by magic when the vinegar passed over the survace of the uterus and the vagina. He believed that the salts of iron should seldom or never be used, but in a case, which was supposable, in which the vinegar failed he would resort to that remedy.

Dr. John T. Atlee, of Lancaster, believed the hand and arm were the most ready and efficient remedy at our command for the arrest of post-partum hemorrhage.

Dr. A. H. Smith thought that post-partum hemorrhage was due to one of two causes, either distention or laceration. If the uterus was emptied it would contract, and the hemorrhage would cease. The main point in the treatment, therefore, was not to introduce anything into the cavity of the uterus which would prevent complete contraction. The means which he had employed with greatest success had been injection of hot water; the water should be injected at the same time the clots were turned out, and at a temperature of 110° F.

Dr. Camabell, of Augusta, Georgia thought the idea of irritating the placental surface was valuable and worthy of consideration. He spoke highly of the use of injections of tr. of iodine. He disapproved of the use of the persalts of iron.

Dr. Englemann, of St. Louis, approved of the use of the iron-salts, and had come to rely upon them in extreme cases.

Dr. Trask, of Astoria, L. I., stated that an analysis of the cases in which iron had been used showed that the benefit which followed was due, not to its styptic, but to its irritant action.

Injections of the tr. of iodine had been his favorite method of treatment.

Dr. Bozeman, of New York, believed that if compression was made over the fundus of the uterus at the same time the hand passed into its cavity to remove the clots, the hemorrhage would almost invariable be arrested.

Dr. J. R. Chadwick, of Boston, thought that a very good remedy in these cases was ether used hypodermically. He had employed it in three cases with the effect of producing prompt tonic contraction.

Dr. Baker, of New York, mentioned the hemorrhagic diathesis as one of the predisposing causes, and related a case in illustration. Because of the existence of this diathesis the woman nearly lost her life, notwithstanding the most vigorous prophylactic treatment. With reference to treatment of this form of hemorrhage he would, if possible, avoid introducing the hand into the cavity of the uterus, because o[illegible] the liability to do injury by such proced[illegible]e.

Dr. [illegible]ilson thought injury to the uterus was not likely to follow introduction of the hand when all the parts were relaxed. He had never used the persalts of iron and did not approve of their use. His hand was the therapeutical agent upon which he relied most, and he had never met with any injuries from its use that confirmed Dr. Baker's fears.

---

Arsenic and its Preparations in the Treatment of Skin Diseases.—Molinari, in an article on this subject (Gaz. Med. Ital:-Lomb., March 1877; Four. des Sci. Med., 1878, p. 100), concludes as follows: 1. Arsenical preparations should be administered at first in small doses, which are to be increased gradually, their offect being carefully watched. At the first sign of disturbance, as loss of appetite, pain in stomach, dryness of the mouth, swelling of the eyes or nose, or difficulty in urination, the medicine should be suspended, but renewed again when these disappear. 2. A saline purgative, as sulphate of sodium, shonld be taken before the beginning of the treatment, and at its close. 3. Arsenic should not be given after, but before, meals: it is better tolerated under these circumstances, and is more quickly absorbed. Acids should not be taken by the patient, and alchoholic drinks only rarely. The treatment should last from one month to six weeks. 4. External means, as ointments, etc., should be added to the arsenical treatment. 5. In eczematous affections, where the kidneys are involved, some diuretic, as the acetate of potassium, may be added to the arsenic, but uot substituted for it.

---

*Chronic chills.*—Dr. Hervey (in *Lancet & Clinic*) suggests the following as a successful treatment to prevent or break up the chill habit in chronic cases:

R. Equal parts of tincture iodine, tincture ferri chloridi., and tinct. sanguinaræ; of this I give from fifteen to thirty drops after each meal.

With this one grain of quinine is given before each meal for the time specified above, ten days after the chills have been broken or other paroxysms arrested. Then I continue the iodine for ten, fifteen, or thirty days longer, until all enlargement of the spleen is removed. I have been treating intermittent fever after this plan, for fully thirty years.

Dr. Kessler (in *Brief*) says:

Living in a malarial district, I am compelled to use all the different remedies at my command; but for simple intermittent fevers, I have never yet failed to get satisfactory results from the following, for a child 5 to 8 years old:

| | | |
|---|---|---|
| R. | Sul. cinchonidia | 30 grains. |
| | Tinc. ferri. chl | 30 drops. |
| | Aqua distil | 1 ounce. |
| | Tinc. nux. vom | 10 drops. |
| | Podophyllin | 2 grains. |
| | Syr. simp. q. s. fiat | 4 ounces. |

M. S. Teaspoonful four times a day; increase dose for older persons.

